# Antibacterial and anti-inflammatory properties of host defense peptides against *Staphylococcus aureus*

**DOI:** 10.1016/j.isci.2022.105211

**Published:** 2022-09-24

**Authors:** Leonardo Cecotto, Kok van Kessel, Margreet A. Wolfert, Charles Vogely, Bart van der Wal, Harrie Weinans, Jos van Strijp, Saber Amin Yavari

**Affiliations:** 1 Department of Orthopedics, University Medical Center Utrecht, 3508GA Utrecht, the Netherlands; 2 Department of Medical Microbiology, University Medical Center Utrecht, 3508GA Utrecht, the Netherlands; 3 Department of Chemical Biology and Drug Discovery, Utrecht University, 3508GA Utrecht, the Netherlands; 4 Department of Biomechanical Engineering, Delft University of Technology, 2628CD Delft, the Netherlands; 5 Regenerative Medicine Centre Utrecht, Utrecht University, 3508GA Utrecht, the Netherlands

**Keywords:** Immunology, Microbiology

## Abstract

Cationic host defense peptides (HDPs) are a promising alternative to antibiotics in the fight against *Staphylococcus aureus* infections. In this study, we investigated the antibacterial and immunomodulatory properties of three HDPs namely IDR-1018, CATH-2, and LL-37. Although all three HDPs significantly inhibited LPS-induced activation of human macrophages, only CATH-2 prevented *S. aureus* growth. When applied to different infection models focused on intracellularly surviving bacteria, only IDR-1018 showed a consistent reduction in macrophage bacterial uptake. However, this observation did not correlate with an increase in killing the efficiency of intracellular *S. aureus*. Here, we conclude that despite the promising antibacterial and anti-inflammatory properties of the selected HDPs, macrophages’ intrinsic antibacterial functions were not improved. Future studies should either focus on combining different HDPs or using them synergistically with other antibacterial agents to improve immune cells’ efficacy against *S. aureus* pathogenesis.

## Highlights

• HDPs namely IDR-1018, CATH-2, and LL-37 inhibit macrophage LPS-mediated activation
• Only CATH-2 shows direct antibacterial properties against *S*. *aureus*
• Reduction in the number of phagocytosed bacteria is rendered by IDR-1018
• HDPs fail to stimulate macrophage’s ability to kill intracellular *S. aureus*

## Graphical abstract

**Figure:**
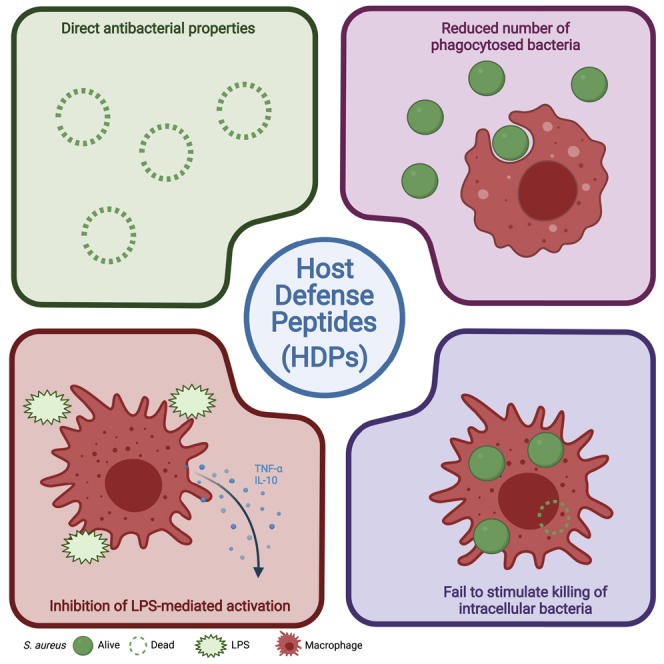

## Introduction

Bacterial infections are one of the most frequent and severe complications associated with the use of biomaterials (Arciola et al., 2018). Despite the significant improvement in medical and surgical management, infection incidence still arises up to 5% after orthopedic surgeries (Moriarty et al., 2016). The majority of biomaterials infections are caused by *Staphylococci*, particularly by *S. aureus* (Campoccia et al., 2006). Over the years, traditional antibacterial strategies became less and less effective against *S. aureus* infections owing to its ability to build resistance to antibiotics and evade immune system recognition and killing mechanisms (de Jong et al., 2019; Flannagan et al., 2016; Horn et al., 2018). Moreover, *S. aureus* can invade, survive, and proliferate inside numerous cell types besides immune cells (Fraunholz and Sinha, 2012; Horn et al., 2018; Strobel et al., 2016), even reaching the narrowest and deepest spaces of the osteocytes canaliculi network (de Mesy Bentley et al., 2017; Muthukrishnan et al., 2019). Finally, *S. aureus* pathogenesis is exacerbated in the presence of biomaterials because they offer an ideal substrate for bacterial adhesion and biofilm formation (Souza et al., 2021).

Despite the progress in treatment efficiency by releasing drugs locally (Amin Yavari et al., 2020; Souza et al., 2021; Wassif et al., 2021), therapeutic compounds able to completely overcome pathogen defense mechanisms and survival are still missing (Abed and Couvreur, 2014; Kamaruzzaman et al., 2017). As we have previously shown, to enhance the therapeutic outcome, implant bio-functionalization strategies should shift focus from antibiotics that eradicate bacteria to enhance host cell response, aiming to improve intrinsic immune cell functions against pathogens invasion (Amin Yavari et al., 2020).

Cationic host defense peptides (HDPs) are naturally occurring molecules participating in the innate immune response in almost all vertebrates. The cathelicidins family of HDPs is the one characterized by a conserved “cathelin” domain with high interspecies homology (Kościuczuk et al., 2012). These molecules are generally 10 to 50 amino acids long and positively charged with amphipathic properties. These features enhance peptide interactions with negatively charged membranes of both bacterial and host cells. Thereby, HDPs could control bacterial infections via two routes: direct antimicrobial activity and regulation of immune response (Hancock and Sahl, 2006). Moreover, the use of HDPs as a potential alternative to antibiotics gained interest thanks to their very low microbial resistance development (Mookherjee et al., 2020).

Among the numerous natural and synthetic HDPs described in the literature, we narrowed down the selection to three well-known cathelicidins: human LL-37, chicken CATH-2, and bovine-derived IDR-1018. These peptides retain broad-spectrum antibacterial activity by direct killing mechanisms, like CATH-2 (Coorens et al., 2017a), or by anti-biofilm and indirect bactericidal properties, such as IDR-1018 and LL-37 (Alford et al., 2021; Biswas et al., 2021; Kang et al., 2019; Reffuveille et al., 2014). Several studies reported these peptide immunomodulatory functions as well. In fact, all three peptides modulate immune cells cytokines production by stimulating chemokine release and inhibiting LPS-mediated activation (Choe et al., 2015; Coorens et al., 2017a; Hancock et al., 2016; Mookherjee and Hancock, 2007; van Dijk et al., 2009). LL-37 promotes the internalization and intracellular killing of pathogens via an increase in ROS production, both in neutrophils (Alalwani et al., 2010) and macrophages (Tang et al., 2015; Ugarova et al., 2016; Wan et al., 2014). IDR-1018 contributes to the neutrophils activation and production of HDPs, including LL-37 (Niyonsaba et al., 2013). Stimulation with IDR-1018 drives macrophages phenotype to an intermediate state, enhancing both pro-inflammatory stimuli against pathogens and pro-healing properties (Pena et al., 2013). Moreover, *in vivo* wound healing improvements have been reported for both IDR-1018 and LL-37 (Ramos et al., 2011; Steinstraesser et al., 2012).

Nevertheless, HDPs’ efficacy against bacterial infection turned out to be controversial among different research groups. Particularly, changes in testing conditions might yield different effects from the same peptide. For instance, peptides’ immunomodulatory properties have been described mainly on non-human cell lines, which can hide possible species- or cell-specific effects (Coorens et al., 2017a). At the same time, HDPs antibacterial properties have been mainly monitored in non-physiological conditions which are different compared to the *in vivo* scenarios (Bowdish et al., 2005; Coorens et al., 2017a; Mookherjee et al., 2020). For this reason, we aimed to characterize and compare IDR-1018, CATH-2, and LL-37 immunomodulatory and antibacterial properties under the same conditions *in vitro*. Furthermore, we tested the ability of single peptides to control *S. aureus* infection either by direct killing or modulating primary human macrophage functions, with a particular focus on pathogens’ intracellular survival.

## Results

### Only CATH-2 had direct antibacterial properties

**Figure 1:**
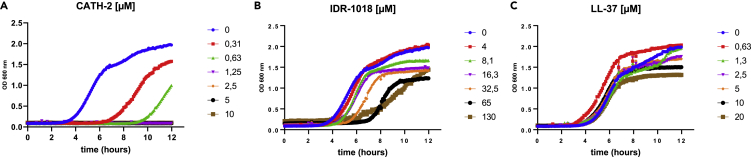
Only CATH-2 had direct antibacterial properties *S. aureus* growth was monitored by measuring OD (600 nm) continuously during 12 h. Bacteria were incubated with a concentration range (given in μM) of peptides of CATH-2 (A), IDR-1018 (B), and LL-37 (C). (n = 3). Data were plotted with mean only.

To evaluate the antibacterial properties of each peptide, we continuously monitored the growth of *S. aureus* in the presence of different peptide concentrations over a period of 12 h. Figure 1A shows that only CATH-2 had direct antibacterial properties. Interestingly, CATH-2 arrested *S. aureus* growth at concentrations 10-times lower than those needed to inhibit macrophage LPS-mediated activation. Both IDR-1018 and LL-37 did not show antibacterial effects at any concentration tested (Figures 1B and 1C). However, IDR-1018 was able to delay the start of *S. aureus* exponential growth only at concentrations starting at 65 μM.

### IDR-1018, CATH-2, and LL-37 inhibited macrophage lipopolysaccharide-mediated activation

**Figure 2:**
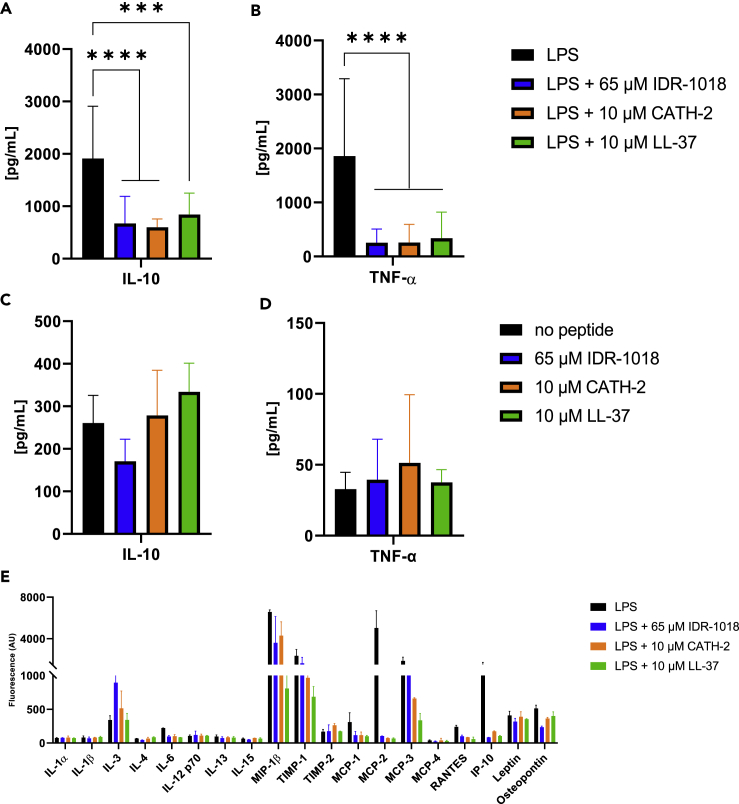
All peptides inhibited macrophage LPS-mediated activation Macrophages were incubated for 24 h with LPS and peptides (A, B), or peptides alone (C, D), and TNF-α and IL-10 levels were quantified by ELISA. (n = 9, from a total of 3 independent experiments). (E) Qualitative expression of several other cytokines was determined by cytokine array kit after 24 h stimulation with LPS and peptides. Fluorescence values are expressed as arbitrary units (AU). (n = 2). Data were represented as +/− SD. One-way ANOVA was used to determine statistical significance. ∗∗∗∗p < 0,0001, ∗∗∗p = 0,0002.

To evaluate the potential anti-inflammatory action of each peptide, we measured the release of TNF-α and IL-10 after the LPS stimulation of primary human macrophages. First, we tested a non-toxic concentration range of each peptide with LPS-stimulated cells. All three peptides efficiently decreased TNF-α and IL-10 release in a dose-dependent manner (Figures S3A–S3F). Subsequently, the optimal concentration of each peptide was tested for both LPS stimulated and non-stimulated cells. As shown in Figure 2, all three peptides inhibited the LPS-induced release of IL-10 (Figure 2A) and TNF-α (Figure 2B) significantly.

At the same time, peptides alone did not trigger a pro-inflammatory response (Figures 2C and 2D). Also, none of these conditions affected the viability of the macrophages as determined by LDH release (Figure S3G). This anti-inflammatory effect of the peptides did not correlate with a clear polarization of macrophages toward an M1 or M2 phenotype, defined by CD80 and CD163 expression, respectively (Figure S4).

To further characterize the potential anti-inflammatory profile of each peptide, expression levels of several cytokines, chemokines, and growth factors were measured after 24 h stimulation with LPS. Overall, it was found that all three peptides altered the LPS-induced expression of several other factors, besides TNF-α and IL-10 (Figure 2E). Moreover, all the peptides limited the over-activation and recruitment of immune cells to the inflammation site by reducing the expression of macrophage inflammatory protein 1β (MIP-1β), RANTES, and monocyte chemoattractant proteins (MCP-1,2,3).

### Use of different infection models to study cationic host defense peptides’ contribution to bacteria phagocytosis

Once the anti-inflammatory and direct antibacterial profile for each peptide was defined, we aimed to explore HDPs’ ability to influence macrophage antibacterial functions via different infection models focusing on bacteria surviving intracellularly. Based on the anti-inflammatory effect of each peptide (Figure 2), macrophages were stimulated with 65 μM IDR-1018, 10 μM CATH-2, or 10 μM LL-37 before infecting them. In all infection models, the same time points after infection were selected: 30 min to determine *S. aureus* uptake by immune cells and 24 h to evaluate macrophage bactericidal activity against intracellular bacteria.

**Figure 3:**
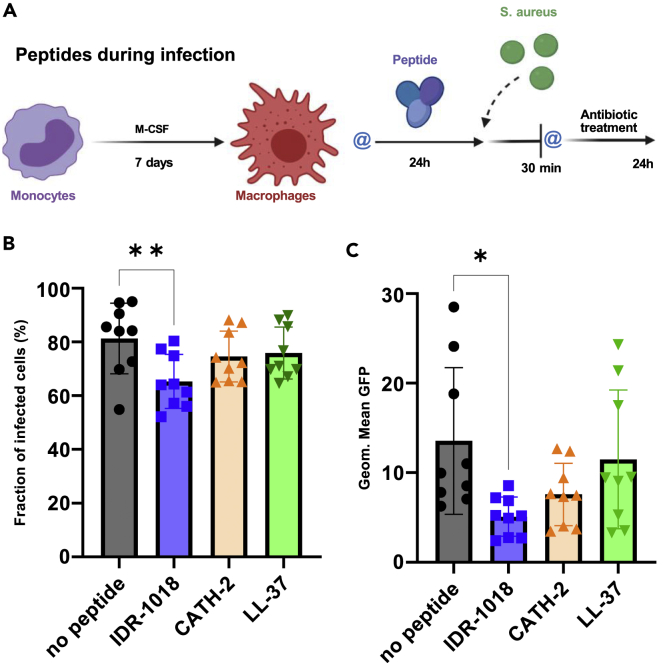
When used during infection, IDR-1018 reduced the number of bacteria phagocytosed by macrophages Macrophages were first stimulated with the peptides and then infected with *S. aureus* as outlined in the “peptides during infection” model (A) (Created with BioRender.com) where @ represents 2 washing steps. Results from the 30 min time point are shown. The percentage of macrophages that had taken up at least 1 bacterium is depicted as the fraction of infected cells (B). The bacterial load is represented as the geometric mean of the GFP signal (C). (n = 9, from a total of 3 independent experiments). Data were represented as +/− SD. One-way ANOVA was used to determine statistical significance. ∗∗p < 0,01, ∗p < 0,02.

As a first approach, macrophages were treated for 24 h with peptides and subsequently infected by *S. aureus* while peptides were kept in the culture media, hence named “peptides during infection” model (Figure 3A). Given the proportion of macrophages that phagocytosed at least 1 bacterial cell, IDR-1018 was the only peptide that significantly reduced the number of infected cells after 30 min (Figure 3B). In addition, according to the geometric mean of GFP signal intensity that verifies the number of intracellular bacteria, only IDR-1018 was able to markedly decrease phagocytosis, while CATH-2 slightly reduced the bacterial load in macrophages (Figure 3C). After 24 h, a similar trend was observed with IDR-1018 as the only peptide that reduced the amount of phagocytosed *S. aureus* (Figure S5).

**Figure 4:**
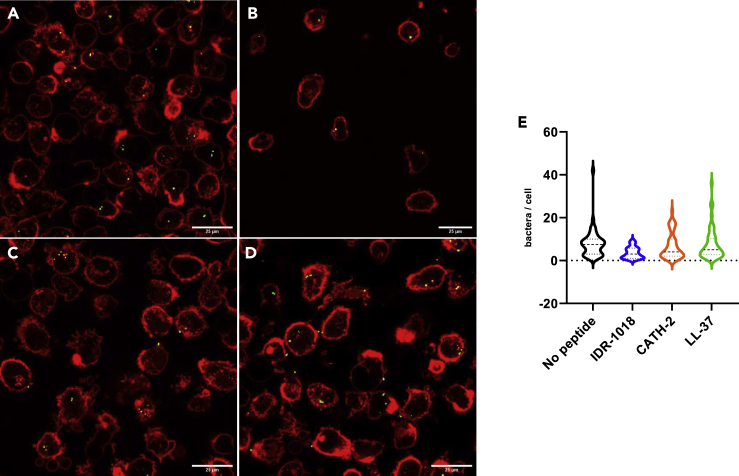
Confocal images confirmed the flow cytometry observations Macrophages were incubated without peptide (A), with IDR-1018 (B), CATH-2 (C), or LL-37 (D). After 30 min infection with *S. aureus* (green dots), cells were collected and their membranes stained with Alexa Fluor 647-labeled WGA (in red) for confocal imaging. The number of intracellular bacteria in each macrophage was manually counted from 50 randomly chosen cells (E). Data were represented with violin plots, lines at mean.

Confocal microscopy imaging also confirmed the flow cytometry results. Particularly, manual counting of intracellular bacteria after 30 min infection showed that IDR-1018 and CATH-2 reduced the bacterial uptake by macrophages, while LL-37 did not provide such effect (Figure 4).

**Figure 5:**
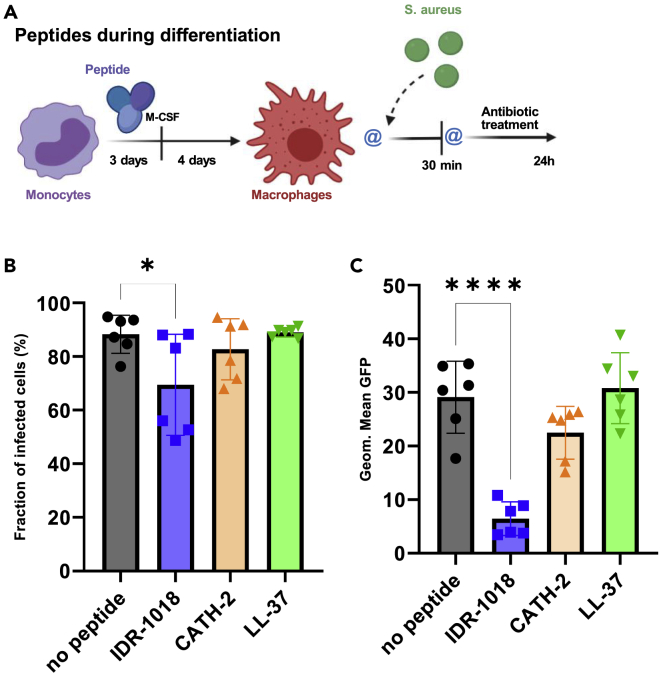
When the peptides were introduced during monocyte differentiation to macrophages, IDR-1018 reduced the number of bacteria phagocytosed by macrophages Before infection, macrophages were differentiated in the presence of each peptide as outlined in the “peptides during differentiation” infection model (A) (Created with BioRender.com) where @ represents 2 washing steps. Results from the 30 min time point showed the fraction of infected cells (B) and geometric mean of the GFP signal (C). (n = 6, from a total of 2 independent experiments). Data were represented as +/− SD. One-way ANOVA was used to determine statistical significance. ∗p < 0,03, ∗∗∗∗p < 0,0001.

Pena and colleagues observed that monocyte differentiation to macrophages in the presence of host defense peptides influenced the mature cell functions (Pena et al., 2013). Here, this aspect was evaluated in the “peptides during differentiation” model (Figure 5A), and the differentiated macrophages were subsequently infected with *S. aureus*. According to Figures 5B and 5C, IDR-1018 significantly reduced both the proportion of infected macrophages and the number of internalized bacteria after 30 min infection. In contrast with the previous “peptides during infection” model, CATH-2 lost its contribution to phagocytosis when it was introduced during the monocyte differentiation.

**Figure 6:**
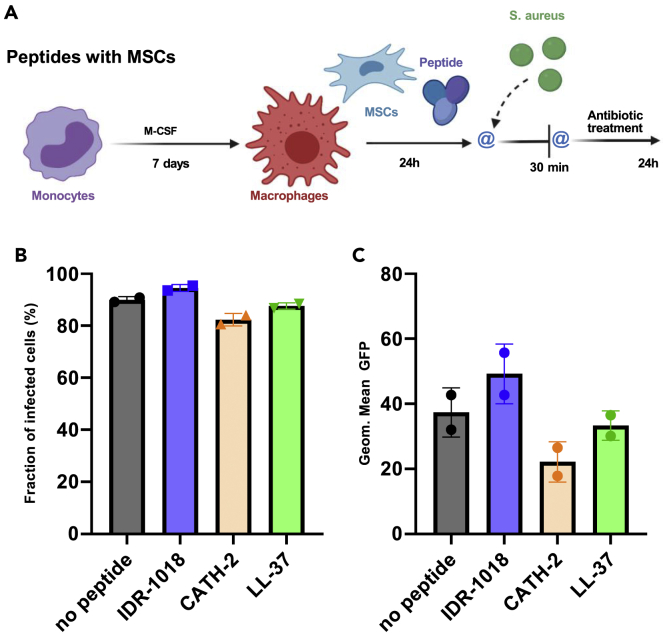
When used in combination with MSCs, none of the peptides influence macrophages’ phagocytosis Macrophages were incubated with MSCs and peptides before infection, as schematized in the “peptides with MSCs” infection model (A) (Created with BioRender.com) where @ represents 2 washing steps. Results from the 30 min time point showed the fraction of infected cells (B) and geometric mean of the GFP signal (C). (n = 2). Data were represented as +/− SD.

In addition to MSCs’ endogenous production of HDPs (*i.e.* LL-37), there are multiple examples of MSCs’ immunomodulatory and antibacterial properties (Alcayaga-Miranda et al., 2017; Chow et al., 2020; Weiss and Dahlke, 2019). Therefore, a combination of different immunomodulatory stimuli was simulated in the “peptides with MSCs” model (Figure 6A), where macrophages were incubated together with MSCs and peptides before infection with *S. aureus*. However, in this scenario, the contribution of the peptides to macrophage phagocytosis was almost nullified, as reported in Figures 6B and 6C.

### Use of different infection models to study cationic host defense peptides’ contribution to intracellular bacteria killing

Furthermore, macrophage bactericidal activity after stimulation with the peptides was investigated. In fact, cells were lysed to quantify the number of viable intracellular bacteria after 30 min and 24 h. To be able to study the intracellular killing capacity of macrophages, all extracellular bacteria and peptides were removed by wash steps and treatment with gentamicin and lysostaphin after 30 min.

**Figure 7:**
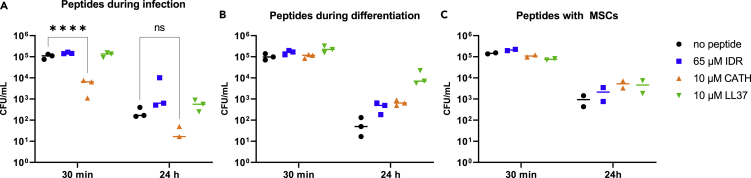
Regardless of the infection models used, none of the peptides reduced the number of bacteria surviving intracellularly Macrophages were stimulated with peptides and infected according to the previously described models. After 30 min and 24 h, cells were lysed and bacteria were enumerated by CFU counting for each infection model. Representative data from peptides during infection (A) (n = 9, from a total of 3 independent experiments), peptides during differentiation (B) (n = 6, from a total of 2 independent experiments), and peptides with MSCs (C) (n = 2). Data were plotted with mean only. One-way ANOVA was used to determine statistical significance. ∗∗∗∗p < 0,0001.

Regardless of the infection model used, non-stimulated macrophages showed an intrinsic ability to kill bacteria surviving intracellularly (Figure 7). In the “peptides during infection” model, CATH-2 significantly reduced the numbers of *S. aureus* surviving intracellularly only after 30 min, while the reduction in CFU after 24 h was not statistically significant. However, no effects were observed for IDR-1018 and LL-37 (Figure 7A).

No change in macrophage-killing properties was observed for all the peptides in both “peptides during differentiation” (Figure 7B) and “peptides with MSCs” (Figure 7C) infection models. On the contrary, in both models macrophage stimulation with peptides showed an increased amount of *S. aureus* surviving intracellularly compared to controls after 24 h.

## Discussion

HDPs received attention as a promising alternative to antibiotics against bacterial infection thanks to their dual functionalities in controlling infection while modulating immune cells’ functions. It is foreseen that these peptides could be potentially implemented into orthopedic implants’ coatings to prevent implant-associated infections (IAI). So far, several HDPs with immunomodulatory and antibacterial properties have been described in the literature. However, the lack of standardized methods to study their functionalities impedes the use of peptides for the next translational steps. Here, we aimed to directly compare the immunomodulatory and antibacterial functions of IDR-1018, CATH-2, and LL-37 through the same *in vitro* conditions.

### Direct antibacterial effect

Although the mechanisms of antibacterial properties for several HDPs have been described (AlMatar et al., 2018; Mookherjee et al., 2020; Sancho-Vaello et al., 2020; Scheenstra et al., 2019; Wieczorek et al., 2010), little is known on peptides interactions with gram-positive bacteria, particularly with *S. aureus*. Schneider et al. showed that CATH-2 bound *S. aureus* membrane through ionic interactions, causing membrane ruffling and intracellular morphological changes (Schneider et al., 2017). Similar cell shrinking and membrane permeabilization effects, but against a different gram-positive bacterium, have been described for LL-37 (Barns and Weisshaar, 2013). On the other hand, the molecular mechanisms leading to the immunomodulatory effects of HDPs are more complex and not properly verified yet (Choi and Mookherjee, 2012; Mookherjee et al., 2020; Steinstraesser et al., 2011).

In this study, only CATH-2 showed a direct killing effect against *S. aureus* (Figure 1). According to different studies that provided examples of both IDR-1018 (Choe et al., 2015; Wieczorek et al., 2010) and LL-37 (Dürr et al., 2006; Noore et al., 2013) bactericidal action, yet the culture conditions highly influenced HDPs direct antibacterial activity (Bowdish et al., 2005; Coorens et al., 2017a; Mookherjee et al., 2020). It should be noted that in all these studies, the peptides’ minimum inhibitory concentration (MIC) was reported differently. This clearly stemmed from the variation in the experimental setup and bacterial strain tested. In addition, Durr et al. showed that bacterial killing and immune cells cytotoxicity of LL-37 were rendered at the same concentrations which undermined its broad-spectrum antimicrobial properties (Dürr et al., 2006).

### Anti-inflammatory effect

All the peptides showed a similar inhibitory action against LPS-mediated activation of macrophages, although higher concentrations of IDR-1018 were required as compared to CATH-2 and LL-37 (Figures 2A and 2B). In addition to TNF-α and IL-10, the peptides affected the expression of several other cytokines involved in immune cells’ activation and recruitment at the inflammation site (Figure 2E). To illustrate, the expression of several pro-inflammatory cytokines, such as MIP-1β, RANTES, and MCP-1,2,3 was reduced, confirming a stronger inhibition of the LPS activation of macrophages by LL-37 (Scheenstra et al., 2019). Furthermore, IL-3 expression, a basophil growth factor also involved in infection-induced response of immune cells (Siracusa, 2016), was up-regulated by all peptides stimulation. In conclusion, this suggests that the selected peptides from one side dampen excessive inflammatory stimuli, while in parallel providing immune cells tools to resolve inflammation. A similar concept was pointed out by Pena et al. where IDR-1018 stimulation did not correlate with the polarization of macrophages toward a clear M1 or M2 phenotype (Pena et al., 2013). Similarly, we observed that also CATH-2 and LL-37 kept macrophages in an intermediate state, between a pro- and anti-inflammatory phenotype (Figure S4).

### Indirect antibacterial effect

*S. aureus* finds protection against most antibiotics and host defenses by hiding inside the host cells. At the same time, infected cells circulating in the bloodstream are used as a “Trojan horse” by the pathogens to spread throughout the body (Thwaites and Gant, 2011). Direct targeting of intracellular bacteria still remains a challenge (Bongers et al., 2019; Liu et al., 2020); therefore, we used HDPs to improve macrophage intrinsic ability to kill intracellular bacteria.

When immune cells were stimulated with the peptides right before and during infection, only IDR-1018 reduced the proportion of cells that phagocytosed bacteria (Figures 3B and 3C). Nevertheless, this did not correlate with a lower number of pathogens surviving within macrophages after 24 h (Figure 7A). Furthermore, CATH-2 only decreased the amount of intracellular *S. aureus* after 30 min, but not significantly after 24 h. Additionally, as bacteria and peptides were incubated together, it is more likely that a decrease in the intracellular bacteria was caused by the direct killing effect of CATH-2 (Figure 1A). This could also explain the flow cytometry results (Figure 3C) which showed a decrease in the number of bacteria taken up by macrophages. Besides, the bactericidal capacity of CATH-2-stimulated immune cells was comparable to the non-stimulated group after 24 h (Figure 7A), which reflected no changes in the macrophages’ killing functions.

In line with the trained immunity theory introduced by Netea and colleagues (Netea et al., 2020), we studied the effect of peptides during monocyte differentiation. In this context, IDR-1018 immunomodulatory function was preserved by mature macrophages, which led to a reduced proportion of infected cells (Figures 5B and 5C). However, even in this case, IDR-1018 had no impact on immune cells’ killing efficiency (Figure 7B). Similarly, monocytes’ differentiation in the presence of CATH-2 and LL-37 had no impact on mature cells’ functions, nor on phagocytosis or intracellular killing.

In our multicellular *in vitro* model, consisting of macrophages cultured together with MSCs, we could not observe any indirect antibacterial effect derived from peptides stimulation. No difference among groups was observed both in terms of the proportion of infected cells (Figures 6B and 6C) and *S. aureus* intracellular survival (Figure 7C). Despite the multiple advantages described by the direct or indirect culture of macrophages and MSCs (Alcayaga-Miranda et al., 2017; Chow et al., 2020; Lu et al., 2021; Weiss and Dahlke, 2019), one might speculate that MSCs interfere with peptides immunomodulatory effects, yet further studies are needed to validate this hypothesis.

As we aim to find valuable therapeutic agents in human treatment, we selected primary immune cells as a benchmark for our experiments. Therefore, we could not verify previous studies that showed LL-37 promoting clearance of intracellular *S. aureus* in a macrophage human cell line (Tang et al., 2015) and bacterial phagocytosis in a macrophage murine cell line (Ugarova et al., 2016). This discrepancy might be ascribed either to the cell-type specificity of peptides or differences in behavior between primary cells and cell lines from different species (Andreu et al., 2017; Fransen et al., 1986; McCarron et al., 2015; Mestas and Hughes, 2004). On the other hand, the bactericidal properties of CATH-2 on the human macrophages, to our knowledge, have not been explored yet.

Together with macrophages, neutrophils play a central role in the first response against invading pathogens. However, neutrophils were not used in this study as their shorter life span makes them a less favorable candidate for *S. aureus* intracellular survival compared to macrophages (Summers et al., 2010; Thwaites and Gant, 2011). Nonetheless, it has been shown that both IDR-1018 and LL-37 improved neutrophils’ antibacterial functions. For instance, IDR-1018 enhanced the killing of intracellular *Escherichia coli* (Niyonsaba et al., 2013), LL-37 improved neutrophils ROS production, and *S. aureus* uptake (Alalwani et al., 2010). However, they have not studied a correlation between the higher ROS produced and intracellular killing.

### Future outlook

The use of HDPs as one of the most promising alternatives to antibiotics has been receiving much attention recently thanks to their influence on both host and bacterial cells, as well as lower risk in developing bacterial resistance (Bagheri et al., 2009; Ferreira and Zumbuehl, 2009; Willcox et al., 2008). Here, the anti-inflammatory and antibacterial properties of three HDPs were studied and reported; however, their efficacy could be potentially improved. Particularly, they could be used in combinatorial or synergistic strategies with each other or other conventional antibacterial agents. For instance, the combination of HDPs with antibiotics (Pletzer et al., 2018; Zharkova et al., 2019), other HDPs (Yu et al., 2016), or other immune cell components (Doolin et al., 2020) already showed improved antibacterial efficiency compared to when the components were used alone. Alternatively, modification to HDPs’ sequence also showed improved immunomodulatory and antibacterial effects (Koro et al., 2016; Narayana et al., 2019; van Harten et al., 2022; Wang et al., 2017). On the other hand, loading HDPs into antibacterial coatings should be considered new local drug delivery strategy to prevent IAI (Amin Yavari et al., 2020; Onaizi and Leong, 2011).

### Conclusions

In this work, the immunomodulatory and antibacterial properties of IDR-1018, CATH-2, and LL-37 peptides were studied under the same *in vitro* conditions. Although the strong anti-inflammatory properties of all peptides were verified, they did not improve macrophages’ antibacterial functions. In fact, only CATH-2 showed promising direct antibacterial properties against *S. aureus*. Furthermore, it was shown that IDR-1018 influenced macrophages’ phagocytosis ability by reducing the number of engulfed bacteria. However, none of the tested peptides enhanced macrophage’s ability to kill intracellular *S. aureus*.

### Limitations of the study

One may argue that the inhibition of LPS-mediated activation of macrophages was not caused by peptides’ direct interaction with the immune cells. It was shown via a mechanism named “silent killing” that CATH-2 and LL-37 bind LPS to inhibit macrophages receptors activation (Coorens et al., 2017b). On the contrary, IDR-1018 did not show significant binding affinity to LPS (Wieczorek et al., 2010).

As peptides decrease LPS-induced cytokines production in a dose-dependent manner, one should investigate the antibacterial properties of macrophages similarly. Any changes in the infection models, such as MOI, time points, presence of peptides during infection, and so forth, may alter the outcomes as well. At the same time, the use of different monocytes’ isolation techniques and differentiating factors may influence the phenotype and functions of mature macrophages (Ambarus et al., 2012; Nielsen et al., 2020).

## STAR★Methods

### Key resources table

**Table:** 

REAGENT or RESOURCE	SOURCE	IDENTIFIER
**Antibodies**		

APC Mouse Anti-Human CD14	BD	Cat#555399; RRID:AB_398596
FITC anti-human CD3 Antibody	BioLegend	Cat#300440; RRID:AB_2562046
FITC Mouse Anti-Human CD19	BD	Cat#555412; RRID:AB_395812
CD15 FITC	BD	Cat#332778; RRID:AB_2868627
FITC anti-human CD16 Antibody	BioLegend	Cat#360716; RRID:AB_2563071
PE/Cyanine7 anti-human CD80 Antibody	BioLegend	Cat#305217; RRID:AB_1877254
APC anti-human CD163 Antibody	BioLegend	Cat#333609; RRID:AB_2291272
Wheat Germ Agglutinin, Alexa Fluo 647 Conjugate	Invitrogen	Cat#W32466

**Bacterial and virus strains**		

SH1000	Gift from Prof. Simon Foster, University of Sheffield	N/A

**Biological samples**		

Macrophages isolated from blood monocytes	Dutch blood bank (Sanquin, Amsterdam, the Netherlands)	N/A
MSCs isolated from bone marrow	Bone marrow from consenting patients in UMC Utrecht	N/A

**Chemicals, peptides, and recombinant proteins**		

IDR-1018	CPC Scientific	Cat#IMMO-006
CATH-2	CPC Scientific	Cat#ATMP-011
LL-37	InVivogen	Cat#tlrl-l37
LPS O111:B4 from *E. coli*	Sigma-Aldrich	Cat#L2630
Gentamycin	Serva	Cat#22185.02
Lysostaphin	Bioconnect	Cat#MBS635842
CellTrace Violet Cell Proliferation Kit, for flow cytometry	Invitrogen	Cat#C34557
SYTOX Orange Dead Cell Stain, for flow cytometry	Invitrogen	Cat#S34861
Trypsin-EDTA (0.25%), phenol red	Gibco	Cat#25200056
Recombinant Human M-CSF	Peprotech	Cat#300-25
Poly-L-lysine solution	Sigma-Aldrich	Cat#P4707
Hyclone fetal bovine serum (HyFBS)	Biowest	Cat#HYCLSV30160
Fetal bovine serum (FBS)	Biowest	Cat#S181H
Human IL4 protein	Biorbyt	Cat#orb80061
MEM α, no nucleosides	Gibco	Cat#22561021
Ficoll Paque Plus	Cytiva	Cat#GE17-1440-02

**Critical commercial assays**		

Cytotoxicity Detection Kit Plus (LDH)	Sigma-Aldrich	Cat#4744934001
Human TNF-alpha DuoSet ELISA	R&D Systems	Cat#DY210
Human IL-10 DuoSet ELISA	R&D Systems	Cat#DY217B
Human Cytokine Array G5	Raybiotech	Cat#AAH-CYT-G5

**Software and algorithms**		

Prism 9	Graphpad	N/A
FlowJo v.10.1	FlowJo LLC	N/A
Fiji	ImageJ	N/A

**Other**		

CELLview Slide	Greiner Bio-One	Cat#543079
CD14 MicroBeads, human	Miltenyi Biotec	Cat#130050201

### Resource availability

#### Lead contact

Further information and requests for resources and reagents should be directed to and will be fulfilled by the lead contact, Saber Amin Yavari (s.aminyavari@umcutrecht.nl).

#### Materials availability

This study did not generate new unique reagents. The authors declare that all data supporting the findings of this study are available within the article and its supplemental information files or are available from the authors upon request.

### Experimental models and subject details

#### Human monocyte-derived macrophages culture

Blood from healthy human donors was supplied by the Dutch blood bank (Sanquin, Amsterdam, The Netherlands). Peripheral blood mononuclear cells (PBMCs) were isolated from buffy coats using Ficoll-Paque density centrifugation. Monocytes were positively selected by magnetic-activated cell sorting (MACS) with anti-CD14 labelled microbeads according to manufacturer instructions.

Isolated monocytes were seeded in a 24-well plate at a density of 300,000 cells/well, except where otherwise stated. Monocytes were differentiated to macrophages by culture for 7 days in α-Minimum Essential Medium (α-MEM) supplemented with 10% (v/v) hyclone fetal bovine serum (hyFBS), 100 U/mL penicillin- streptomycin (1% p/s), and 40 ng/mL human recombinant M-CSF. Culture media was refreshed after 3-4 days.

Viability of the isolated cells was above 75% as determined by Sytox Orange dead cell stain for flow cytometry, before and after differentiation. Purity of the isolated monocytes was above 90% as checked by staining for CD14 and contamination by T-cells (CD3), B-cells (CD19), or granulocytes (CD15) (Figure S1). Cells staining was performed as described in “Peptides influence on macrophage phenotype markers” section, having all fluorochrome-conjugated antibodies diluted 1:30 in PBS with 0,1% BSA and 1% (v/v) heat-inactivated human serum.

#### Human mesenchymal stem cells culture

Mesenchymal stem cells (MSCs) were isolated from human bone marrow aspirates that were obtained from consenting patients. Aspiration procedure was approved by the local medical research ethics committee, University Medical Center Utrecht, under the protocols METC 08-001/K and METC 07-125/C.

Aspirates were diluted in PBS, filtered through a 100 μm cell strainer and the mononuclear cell layer was collected after Ficoll-Paque density centrifugation. Approximately 250,000 mononuclear cells were plated per cm^2^ in MSCs expansion medium consisting of α-MEM supplemented with 10% (v/v) heat-inactivated FBS (FBS), 1% p/s, 0,2 mM L-ascorbic acid-2-phosphate (ASAP). Cells starting from passage 3 were used in the experimental setups.

Before culturing together with macrophages, MSCs were fluorescently labelled with CellTrace Violet diluted in Hanks Balanced Salt Solution (HBSS) according to the manufacturer’s instructions for labelling adherent cells. After staining, MSCs were detached with trypsin/EDTA 0,25% and re-seeded at a density of 100,000 cells/well according to experimental setup.

#### Bacterial culture

All experiments used GFP-labelled *Staphylococcus aureus* strain SH1000, transformed with a GFP-expressing plasmid pCM29 to constitutively express GFP, as previously described (Boero et al., 2021). Bacteria were grown overnight in Todd-Hewitt broth (THB) with 10 μg/mL chloramphenicol to reach stationary phase.

#### Peptides

IDR-1018 (sequence VRLIVAVRIWRR-NH2), CATH-2 (sequence RFGRFLRKIRRFRPKVTITIQGSARF-NH2), and LL-37 (sequence LLGDFFRKSKEKIGKEFKRIVQRIKDFLRNLVPRTES) purity (>95%) was verified by the manufacturers via MS and HPLC.

Peptides were diluted at different concentrations in α-MEM supplemented with 10% FBS or in THB when testing their effects on macrophages or bacteria, respectively.

### Method details

#### Peptides direct antibacterial properties

Peptides direct antibacterial properties were determined by broth micro-dilution method. Overnight bacterial suspension was diluted in THB to reach a final inoculum concentration of 5 x 10^5^ colony-forming units per mL (CFU/mL). Bacterial suspension and peptides dilutions were mixed in equal parts in a flat-bottom 96-well plate in triplicates in a total volume of 200 μL, and incubated at 37°C. Bacterial growth was monitored by measuring OD (600nm) continuously every 5 minutes for 12 h in Clariostar plate reader (BMG labtech) with gentle shaking before each measurement.

#### Peptides anti-inflammatory properties and cytotoxicity

Monocytes were seeded in a 96-well plate at a density of 150,000 cells/well. After 7 days differentiation, macrophages were incubated with fresh media containing a range of peptides concentrations alone or in combination with 10 ng/mL LPS O111:B4 from *E. coli*. After 24 h stimulation, the supernatant was collected to measure LDH levels or TNF-α and IL-10 by ELISA, according to the manufacturer’s instructions. Both LDH and ELISA assays were performed in three independent experiments with measurements in triplicate.

Qualitative expression of various cytokines and chemokines was measured in duplicate for selected conditions by human cytokine array G5, according to the manufacturer’s instructions.

#### Peptides influence on macrophage phenotype markers

After 7 days differentiation, macrophages were incubated with fresh media with LPS, IL-4, and optimal concentrations of peptides alone or in combination with LPS. After 24 h stimulation, macrophages were detached from the culture plate and processed for staining into a 96-well plate. All washing steps were performed with cold 0,1% (w/v) bovine serum albumin (BSA)/PBS and centrifugation at 5 min, 500 x g. A panel of surface molecules was selected, based on previous reports for human macrophage polarization (Ambarus et al., 2012). The staining solutions were prepared by diluting the following fluorochrome-conjugated antibodies in PBS with 0,1% BSA and 1% (v/v) heat-inactivated human serum: CD16 (1:50); CD80 (1:50); CD163 (1:50). As a negative control, staining solution without antibodies was used. Cells were incubated with staining solutions for 30 min on ice, in the dark. Markers expression was measured via flow cytometer (FACSVerse, BD) and data analyzed using FlowJo.

#### Peptides indirect antibacterial properties

To assess the peptides influence on macrophages antibacterial properties, three different infection models studying intracellular bacteria survival, as outlined in Figures 3A, 5A, and 5D, were adopted. In all models, selected concentrations of peptides were used: 65 μM IDR-1018; 10 μM CATH-2; 10 μM LL-37.

The first step concerned the “peptides during infection” model, where cells were washed twice before incubation with the peptides for 24 h and subsequently bacteria were directly added to the culture without any washing step. In a subsequent step we assessed the “peptides during differentiation”, where peptides were added to the differentiation media for the first 3 days and removed after the regular media change; cells were washed twice before infection. In the third step we tested “peptides with MSCs”, where macrophages were cultured in presence of MSCs and peptides for 24 h; before infection cells were washed twice. All washing steps, before or after infection, were performed using warm α-MEM.

In all three models described, the same *S. aureus* infection protocol was applied. An overnight bacterial culture was diluted in α-MEM to reach a final inoculum concentration of 1 x 10^7^ CFU/mL, and opsonized in 5% human pooled serum (HPS) for 15 min at 37°C. Opsonized *S. aureus* was added to the culture at a multiplicity of infection (MOI) = 1, meaning 1 bacterium per eukaryotic cell. To synchronize bacterial uptake, plates were centrifuged for 5 min, 110 x g at RT, and then moved to the incubator at 37°C, 5% CO_2_ for additional 25 min or 24 h. To study intracellular bacterial survival, after 30 min the cells were washed twice and cultured in media supplemented with 100 μg/mL gentamicin and 20 μg/mL lysostaphin for 1 h. Afterwards, cells were washed twice and incubated in media with only 5 μg/mL gentamicin. This treatment allows only intracellular bacteria to survive, as both gentamicin and lysostaphin are unable to penetrate mammalian cell membranes within short time periods (Hamza et al., 2013; Hamza and Li, 2014).

Samples were analyzed by flow cytometry and CFU counting at 30 min and 24 h after infection. Cells for flow cytometry were detached from the culture plate using 1mM DPBS/EDTA in combination with gentle scraping. When macrophages were cultured together with MSCs, samples were first trypsinzed and then scraped in DPBS/EDTA if cells were still attached to the bottom of the culture plate. Cells were moved to a 96-well plate and fixed in paraformaldehyde 4% before analysis. Samples were measured with MACSquant VYB (Miltenyi Biotech) flow cytometer and data analyzed with FlowJo. Gating strategy is summarized in Figure S2. Briefly, a total of 10,000 events were collected for each sample gated on the macrophage population based on forward scatter (FSC) and side scatter (SSC) parameters. When cultured with fluorescently-labelled MSCs, the macrophage population was further selected based on the signal of CellTrace Violet. Non-infected samples were used to set GFP fluorescence baseline and define the proportion of infected, GFP-positive cells.

To quantify the numbers of intracellular bacteria, cells were lysed with Triton X 0,1% and then plated on Todd-Hewitt agar plates in serial dilutions. Plates were incubated overnight at 37°C after which colonies were counted.

#### Confocal images

According to the “peptides during infection” model, after 30 min infection cells were harvested and fixed as described for flow cytometry analysis. Cells membranes were stained with 3 μg/mL Alexa Fluor 647-labelled Wheat Germ Agglutinin (WGA) for 10 min at RT, on a shaking plate. Then, samples were transferred to CELLview slide previously coated with poly-L-lysine, and imaged on a Leica TCS SP5 microscope with a HCX PL AP CS 63x/1.40-0.60 OIL objective (Leica Microsystems). For each condition, the number of intracellular bacteria was counted in 50 randomly chosen cells. Images were adjusted for publication using Image J Fiji.

### Quantification and statistical analysis

GraphPad Prism 9 was used to create the graphs and determine statistical significance via one-way ANOVA.

## Data Availability

• The data reported in this paper will be shared by the lead contact upon request. • This paper does not report original code • Any additional information required to reanalyze the data reported in this paper is available from the lead contact upon request.
