# Supplementary material for: Antibacterial and anti-inflammatory properties of host defense peptides against Staphylococcus aureus
Source: iScience. 2022 Sep 24;25(10):105211. doi: 10.1016/j.isci.2022.105211 (PMC9563556; doi:10.1016/j.isci.2022.105211)

iScience, Volume *25*

Supplemental information

Antibacterial and anti-inflammatory properties of host defense peptides against *Staphylococcus aureus*

Leonardo Cecotto, Kok van Kessel, Margreet A. Wolfert, Charles Vogely, Bart van der Wal, Harrie Weinans, Jos van Strijp, and Saber Amin Yavari

SUPPLEMENTAL INFORMATION

<u>Figure S1.</u> Flow cytometry gating strategy to assess monocytes isolation quality. Same gating strategy was applied for monocytes (upper row) and macrophages (lower row). (A) Selection of total cells population (total) in the linear FSC and SSC. (B) Selection of sytox negative cells within the total population. (C) Distribution of sytox negative population (blue) within total cells population (red) recorded by the flow cytometer. (D) Cells within the sytox negative gate were further divided based on their combined expression of CD14 and CD3/CD19/CD15. Related to STAR Methods.

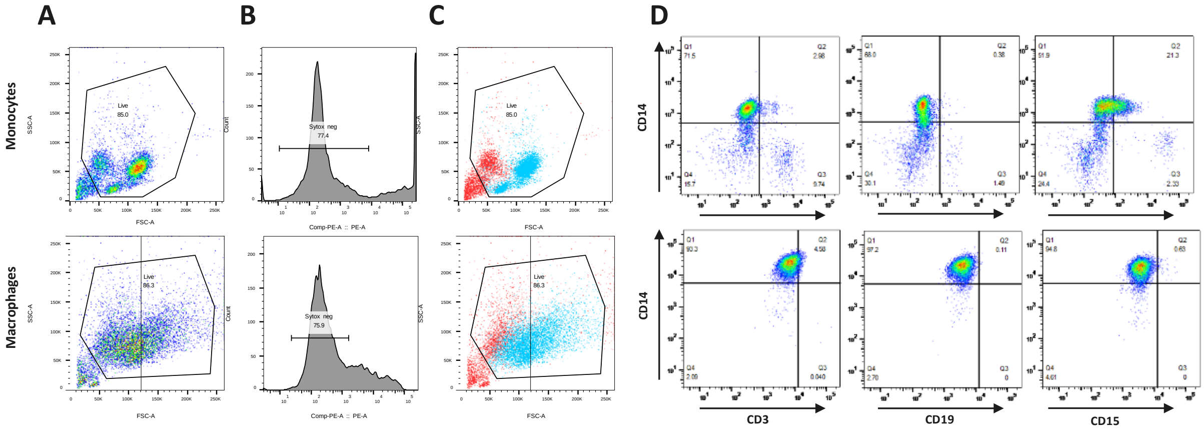

<u>Figure S2.</u> Flow cytometry phagocytosis gating strategy. (A) Selection of macrophage population (live gate) in the linear FSC and SSC. (B) When cultured with MSCs, the macrophage population was further selected based on the signal of CellTrace Violet. (C) Histogram setting GFP fluorescence baseline for non-infected cells. (D) Proportion of non-infected (left peak) and infected (right peak) cells. Related to STAR Methods.

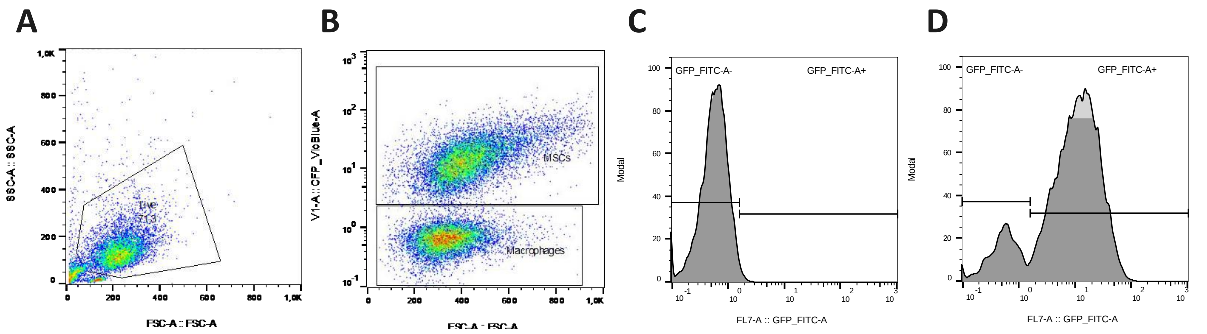

<u>Figure S3</u>. Identification of optimal peptide concentration needed to inhibit macrophages LPS-mediated activation. All peptides tested decreased LPS-induced production of TNF-α (A-C) and IL-10 (D-F) in a dose-dependent manner. (n=3). Any of the selected concentrations of each peptide, in presence or absence of LPS, affected macrophages viability as determined by the levels of LDH released in the culture media (G). (n=9, from a total of 3 independent experiments). Error bars represent SD. Related to Figure 2.

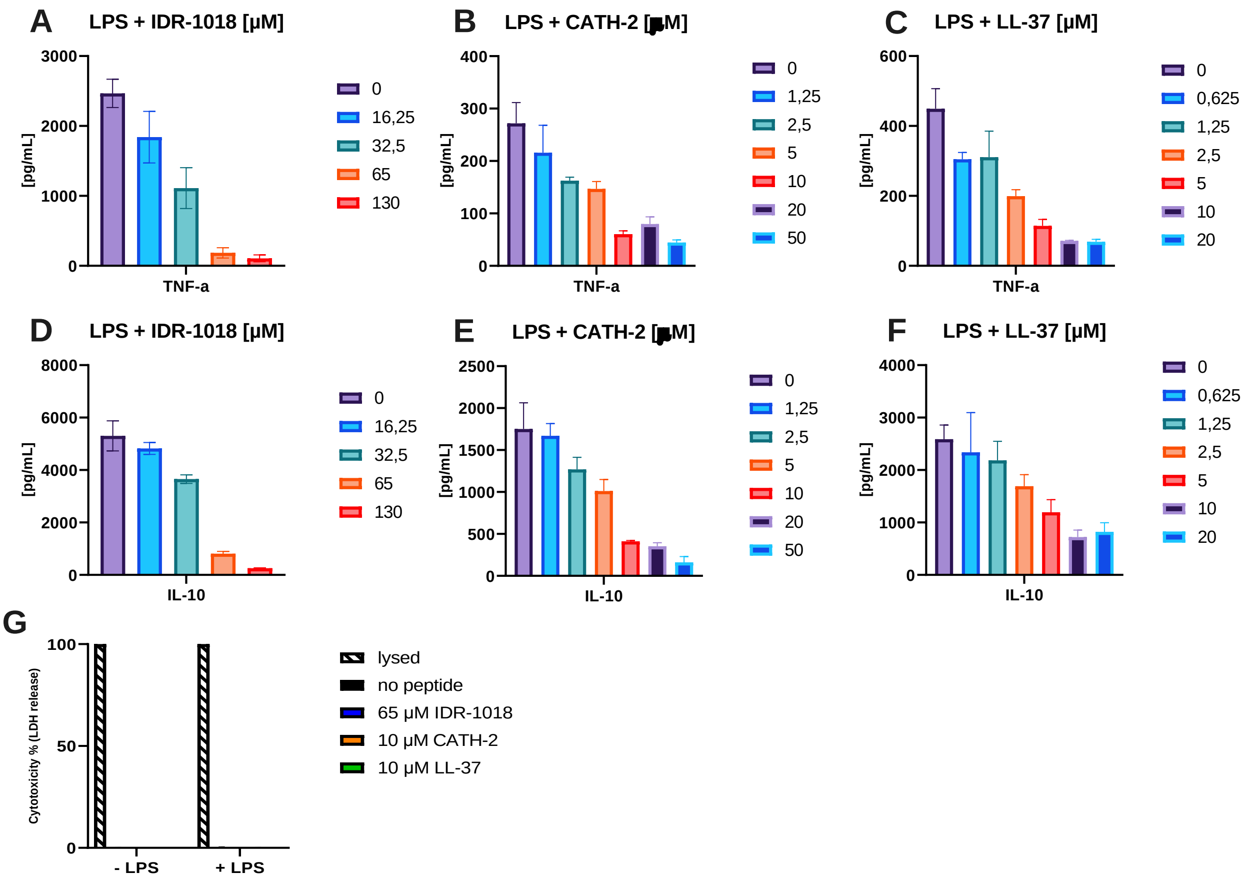

<u>Figure S4</u>. Anti-inflammatory effect of peptides did not correlate to macrophage phenotype polarization. Macrophages were stimulated 24 h with LPS, IL-4, and selected concentrations of each peptide alone (A,B) or in combination with LPS (C,D). Then, macrophage marker expression was measured by flow cytometry to determine macrophage polarization towards a M1 (CD80) or M2 (CD163) phenotype. (n=3). Error bars represent SD. Related Figure 2 and results section "IDR-1018, CATH-2, and LL-37 inhibited macrophage LPS-mediated activation".

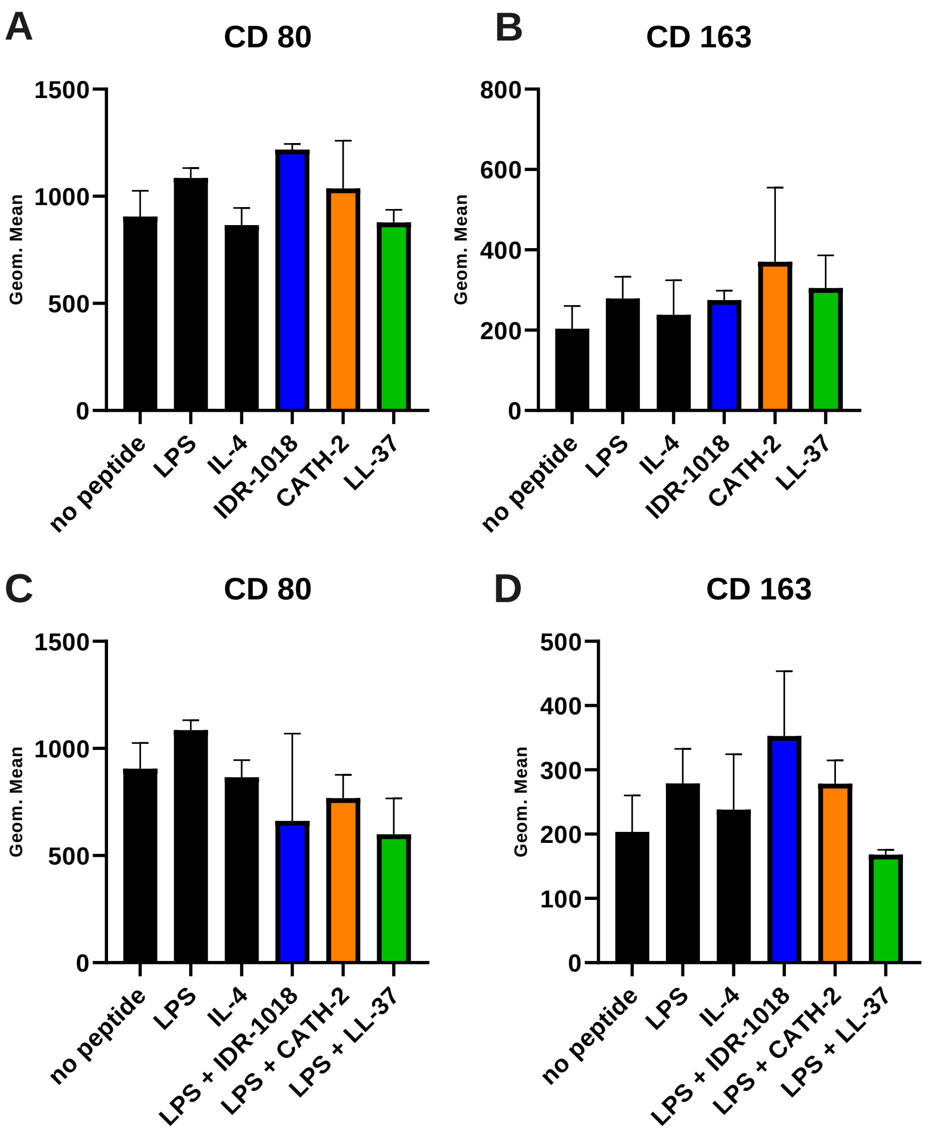

<u>Figure S5</u>. IDR-1018 reduced the number of bacteria phagocytosed by macrophages after 24h. Proportion of infected cells (A) and geometric mean (B) after 24 h infection following the "peptides during infection" model protocol. *p<0,03. (n=9). Error bars represent SD. Related to Figure 3.

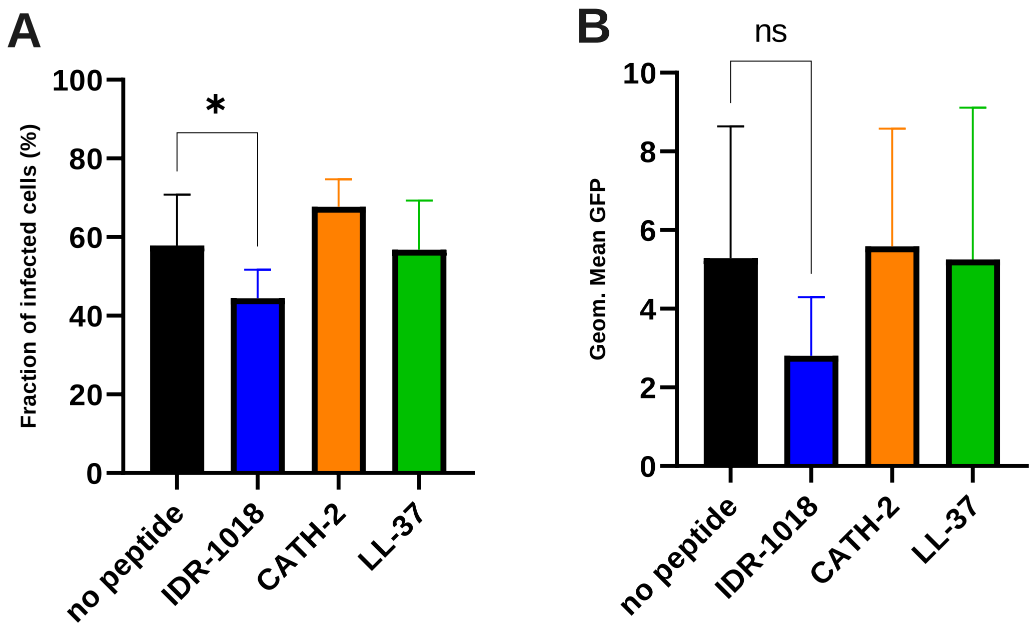

Supplement: Document S1. Figures S1–S5 [file mmc1.pdf]
